# Educational note: types of causes

**DOI:** 10.1093/ije/dyz229

**Published:** 2019-11-09

**Authors:** Neil Pearce, Jan P Vandenbroucke

**Affiliations:** 1 London School of Hygiene and Tropical Medicine, London, UK; 2 Leiden University Medical Center, Leiden, The Netherlands; 3 Department of Clinical Epidemiology, Aarhus University, Aarhus, Denmark

**Keywords:** Epidemiological methods, causal inference, causes

## Abstract

We explore the different types of causes that are commonly investigated by epidemiologists. We first distinguish between causes which are events (including actions) and causes which are states. Second, we distinguish between modifiable and non-modifiable states. This yields three types of causes: fixed states (non-modifiable), dynamic states (modifiable) and events (including actions). Different causes may have different characteristics: the methods available to study them, the types of possible biases, and therefore the types of evidence needed to infer causality, may differ according to the specific cause-effect relationship under study. Nevertheless, there are also substantial commonalities. This paper is intended to improve understanding of the different types of causes, and the different types of causality, that underpin epidemiological practice.

**Key Messages**

- The causes that epidemiologists study can be classified as events or states.
- We can also distinguish between modifiable and non-modifiable states.
- This yields three types of causes: fixed states (non-modifiable), dynamic states (modifiable) and events.
- Different types of causes have different characteristics: the methods available to study them and the types of evidence needed to infer causality may differ.
- There are also substantial commonalities, and all of these types of causes are amenable to epidemiological investigation, causal inference and causal effect estimation.

## Introduction

We need concepts of causality to act in the world in which we live, but it is a notoriously unsettled question as to how we arrive at such concepts in a satisfactory fashion. Historical philosophical discussions about causality include little, if any, consideration of ‘types of causes’. Since John Stuart Mill, it has been standard to admit a plurality of causes for every event^1^ but not to assert that there are many types of causes. Neither did other philosophers do so, to our knowledge, because the need for any distinction was never acknowledged. However, it is evident that the various causes studied by epidemiologists have different characteristics. Some causes can be randomized in practice (e.g. exercise), or in theory (e.g. smoking); some cannot be randomized (e.g. sex, hypertension, genetics), but some of these can be modified with interventions (e.g. interventions to reduce hypertension). Thus, different types of evidence are potentially available to study different causes, and different epidemiological and statistical methods may be available and/or necessary to infer causality.

In this paper, we therefore consider the different characteristics of the different causes that are studied by epidemiologists, and we consider the implications of these differences for epidemiological practice. Our aim is to find a way of describing all of the (reasonable) causal questions that epidemiologists may wish to ask, while acknowledging the differences between the kinds of causal knowledge that we seek and the different methods we might use in attempting to answer these questions.

## Preliminary considerations

### What is a cause?

Philosophers have debated the nature of causality for millennia. The starting point of post-Enlightenment thinking about causation is David Hume, who gave two non-equivalent definitions. The first of these dominated scientific thought until the second half of the 20th century, when there was a remarkable switch in a number of disciplines to the other concept.^2^

The first of Hume’s definitions is as follows.

…we may define a cause to be an object, followed by another, and where all the objects similar to the first are followed by objects similar to the second.

This says that, to be a cause, A has to be constantly followed by B. This is known as a regularity theory or constant conjunction theory of causation. However, Hume’s very next sentence offers a different definition (1748):^3^

Or in other words where, if the first object had not been, the second never had existed.

The phrase ‘in other words’ is incorrect, as many commentators have noted. This distinction between the first and the second definitions of Hume corresponds to the distinction between the positivist (inductive) and the realist (counterfactual) approaches to science.^2^

Hume never followed up his second, counterfactual, definition of ‘cause’, and there was no serious development of the idea that causation might be some kind of counterfactual dependence, until the 1970s. Then, for some reasons that are outside of the scope of this paper, counterfactual thinking was simultaneously explored in philosophy, epidemiology, law, economics and possibly elsewhere too.^2^ This approach revolves around the idea that a cause is something without which the effect(s) would not have happened, and is fundamental to the concepts of causation, and of types of causes, which we explore in this paper.

### Levels of causality

It should be noted that causality can be studied at many levels,^4^ e.g. population factors such as socioeconomic position, individual factors such as ‘lifestyle’, the organ burden of an exposure postulated to be a carcinogen, or the effect of an exposure on DNA methylation that leads to altered gene expression. Understanding disease causation at different levels is useful, as is ascertaining the extent to which the observed effects at one level are explained by known risk factors at other levels.^4^^,^^5^ For instance, tobacco smoke may appear to be a risk factor operating at the individual level. Yet the extent of exposure and susceptibility to exposure may be affected by a wide range of political, economic and social factors. The extent of exposure will be increased if the tobacco industry has unfettered access to poor and under-educated markets, which will lead to a public health problem in developing countries; susceptibility may vary between populations for genetic reasons; and concomitant exposures such as asbestos may affect the risk.^6^ On the other hand, tobacco smoke ultimately has effects at the cellular and molecular levels, which may involve common pathways to other causes of lung cancer.

## Types of causality

As a preliminary, we should first consider a more fundamental level, in which it is accepted that there are two types of causality that epidemiology might be aiming at: the explanatory versus the interventionist. For example, in a commentary on the occasion of the 40th anniversary of the publication of the sufficient cause model by Rothman, VanderWeele writes that:

The sufficient cause framework begins with the outcome or effect to be explained and considers all of its possible causes. Said succinctly, the potential outcomes framework considers the effects of causes, whereas the sufficient outcomes framework considers the causes of effects.^7^

This dovetails with the opinion by Glymour and Glymour that:

There is a counterfactual/interventionist notion of causation—of use when one is designing a public policy to intervene and solve a problem—and a historical, or more exactly, aetiological notion—often of use when one is identifying a problem to solve.^8^

Glymour and Glymour state that one should not try to twist the latter type of causality into an interventionist framework, because it is useful by itself. We do not intend to explore these distinction any further in this paper, but we do wish to note it, since it underlies many current debates about the nature of causality.

## Epidemiological concepts of causation

Epidemiologists have often been uncertain or ambiguous about the nature of causation. To the best of our knowledge, the first definition was by Abraham Lilienfeld in 1957:^9^

A factor may be defined as a cause of a disease, if the incidence of the disease is diminished when exposure to this factor is likewise diminished.

MacMahon and Pugh^10^ defined a cause as:

An association may be classed as presumptively causal when it is believed that, had the cause been altered, the effect would have been changed.

Both descriptions address single causes. In contrast, in 1976, Rothman^11^ introduced a more general model of ‘sufficient’ causal constellations. These are combinations of ‘component causes’ that when acting together are sufficient for disease to occur. In this model, a cause is defined as:

…an act or event or state of nature which initiates or permits, alone or in conjunction with other causes, a sequence of events resulting in an effect. A cause which inevitably produces the effect is sufficient.

A disease will typically have many different causal constellations (combinations of single causes) that would be sufficient causes. Moreover, a particular factor may be a component of more than one of these constellations. For example, in a younger woman the constellation of causes that leads to myocardial infarction may include use of oral contraceptives and smoking. In an older man these might be absent but other factors might come together to produce the same outcome, such as diabetes and hypertension. Rothman^11^ noted that ‘most causes of interest in the health field are components of sufficient causes, but are not sufficient in themselves’. The identification of all the components of a sufficient cause is not necessary for prevention, since blocking the causal role of one component renders a specific sufficient cause insufficient. Philosophically, this approach aligns with the work of Mackie on INUS conditions (**I**nsufficient but **N**on-redundant parts of a condition which is itself **U**nnecessary but **S**ufficient for the occurrence of the effect), since each component of a sufficient cause is usually both insufficient and not necessary, as there might be other constellations of sufficient causes.^12^ Rothman’s framework makes explicit that an exposure will only cause (at least some cases of) a disease if the necessary co-factors are also present. Causation is context-specific, and an exposure may cause disease in some populations, but not in others.^13^

More recently, a direct link between philosophical and statistical thinking about causation was proposed with the work of Judea Pearl.^14–17^ He has proposed three levels of causation.^18^ The first involves observing regularities and essentially corresponds to Hume’s regularity theory (i.e. induction). The second involves deliberate alterations of the environment (i.e. interventions), but can be conceived within a counterfactual framework [if A had (not) been done, would B have occurred?]. The third level of causation is more explicitly counterfactual [if A had (not) been, would B have occurred?].

## Distinction 1: states and events

In this paper, we distinguish between causes which are states (e.g. having the BrCa1 gene mutation), and causes which are events (e.g. smoking). A standard view in philosophy regards the latter activities as events^19–22^; one also finds considerable support for regarding them as facts,^23^^,^^24^ and occasional support for such other entities as features,^25^ tropes,^26^ states of affairs,^27^ situations^28^ and aspects.^29^ We prefer the term ‘events’, since this is broader: for example, an earthquake is an event which can cause death, whereas the use of the terms ‘actions’ or ‘interventions’ implies some human involvement.^30^^,^^31^

There has been a lengthy debate in epidemiology journals as to whether definitions of cause should be limited to events, or can also include states. In particular, there has been considerable debate as to whether states such as ‘race’,^8^^,^^32^^,^^33^ or obesity,^34^ can be regarded as causes and/or whether it is possible to estimate their causal effects. Other examples of states that can be regarded as causes include diabetes as a cause of incident coronary heart disease, hyperthyroidism as a cause of atrial fibrillation, hypertension as a cause of stroke and hypercholesterolaemia as a cause of incident coronary heart disease. Thus, in clinical practice, states are routinely regarded as causes of disease, and many preventive clinical and public health interventions have been developed, e.g. statins to reduce cholesterol and antihypertensives, as well as diet, exercise etc.

The definitions of Lillienfeld and some of the current literature about causality^18^^,^^34^^,^^35^ tend towards the narrower interventionist conception, which is consistent with the historical role of randomized controlled trials (RCTs) in health research,^33^ whereas Rothman and Pearl tend towards the broader aetiological conception. In particular, Pearl’s level 2 causality only applies to events (interventions), but level 3 causation applies to both events and states. This is because Pearl’s level 3 concept of a cause is proposed within a counterfactual framework, and does not specify or restrict the nature of the ‘exposure’. This is exemplified by the fact that it feels natural to represent both states (e.g. having the BrCa1 genetic mutation) and events (e.g. smoking) as causes in a directed acyclic graph (DAG).^36^

In the wake of recent debates, VanderWeele recently proposed a definition of ‘hypothetical intervention’^37^ as the specification of a potentially counterfactual state of exposure in which the ‘state or event’ is sufficiently well specified. This approach allows a well-specified state [e.g. body mass index (BMI) of 35 compared with BMI of 25] to be regarded as a cause. Vanderweele’s approach can be viewed as an attempt to reconcile the two viewpoints, in that states can be accepted as causes since the comparison of two states can be regarded as a hypothetical intervention. Nevertheless, Pearl^36^ criticizes VanderWeele for not being sufficiently explicit in this regard, and thus not inclusive.

In this context, it should be emphasized that regarding a state as a cause is not merely a statistical assumption, but is rooted in biology. Obesity is the state which has perhaps received the most debate as to whether it is a cause, perhaps because it seems a rather ‘fuzzy’ exposure that arises from several different causes (nutrition, lack of exercise etc.) and has several subtypes (brown fat, abdominal fat, etc.). However, there is good biological evidence as to the mechanisms by which the ‘state’ of obesity causes disease; in particular, it involves adiposity, which produces ongoing tissue inflammation and other bodily changes which can result in diabetes, stroke and cardiovascular disease.^38^ Similarly, hypertension produces ongoing challenges to the cardiovascular system which can result in stroke and cardiovascular disease. The fact that these states are causes is reflected in the fact that almost all interventions which modify these states (e.g. gastric surgery, exercise, diet etc.) produce changes in risk which are consistent with those predicted by epidemiological studies of the causal effects of these states. For example, genetic Mendelian randomization studies and intervention studies on hypertension produce reductions in cardiovascular disease risk which are very consistent with the risk estimates from observational cohort studies comparing different levels of hypertension.^39^

In summary, the epidemiological literature has differed over the distinction between the broader conception of cause (‘if the first object had not been’), which might point to an aetiological explanation, and the narrower conception (‘had the cause been altered’), which might point to an intervention. Both are useful and necessary. The narrower view may directly lead to action, but the broader view is consistent with general biological explanations and also with approaches in other areas of science,^14^ many of which study phenomena that are clearly beyond the scope of any intervention (e.g. astronomy or geophysics).

## Different types of causes in epidemiological practice

We will explore the characteristics of these different conceptions of causes by discussing several examples of constellations of different types of causes (Box 1^40^). In Box 1, Factor A and Factor B both affect the risk of disease. People who are exposed to both factors have a higher disease risk than people who are exposed to only one, or to none. The specific examples are discussed in more depth in Box 1. These examples share the characteristic that Factor A is a state (PKU gene, female sex, obesity), whereas Factor B is an event (eating a high phenylalanine diet, sexism, drinking alcohol). Some states (e.g. ethnicity, sex, PKU genes) may only cause disease if other component causes (e.g. racism, sexism, high phenylalanine diet) are also present.

**Box 1. Examples of types of causes**

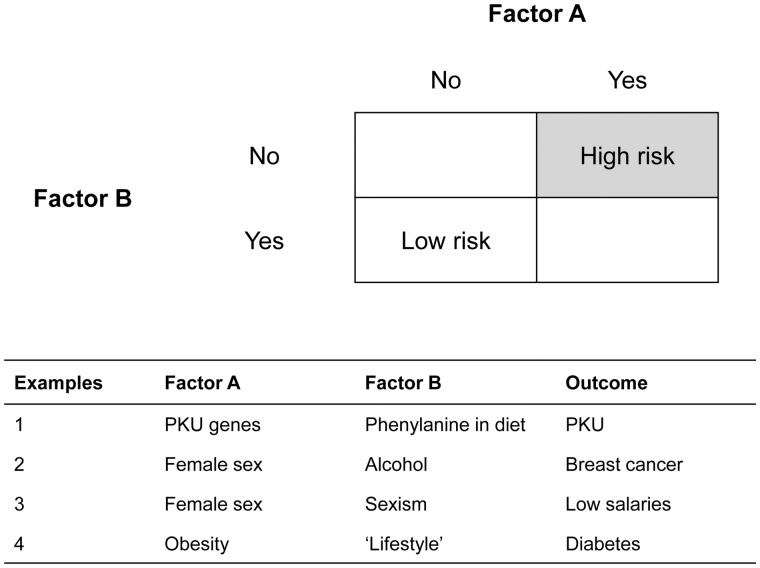

Figure 1 gives several examples in which two factors can each individually, and/or in combination, cause cases of disease.

Example 1 relates to the risk of phenylketonuria (PKU). This is a classic ‘genetic’ disease (albeit one that involves a large number of genes) involving primarily intellectual disability. PKU results from a combination of the PKU genes (Factor A), and a diet high in phenylalanine (Factor B). Each of these factors (having at least one of the PKU genes and a high-phenylalanine diet) is a necessary but not sufficient cause of the disease. Together they form a sufficient cause, i.e. the causal constellation that involves both of these ‘exposures’ is a sufficient causal constellation. We live in a world where almost everyone has a high phenylalanine diet, whereas only a small proportion of people have the relevant polymorphisms of the PKU genes. Thus, the disease appears to be almost entirely genetic, because having the genes is essentially synonymous with developing the disease. Virtually 100% of the population variation is explained by genetics, and almost none of it is explained by diet. Nevertheless, changing the genes is currently impractical, whereas changing the diet can avert the disease. Consequently, PKU is regarded as a classically genetic disease, but the intervention is environmental. This is because PKU is caused by the joint effect of the genes and the high-phenylalanine diet: if either of them is not present (‘if the first object had not been’), then PKU will not occur. However, these two causes are of different types. Having a high-phenylalanine diet is an action (eating!) that occurs several times a day, and which can be conceived of as an intervention (we could randomize people to a high- or low-phenylalanine diet). Having the PKU genes is a ‘state’ that is constant over time.

Example 2 involves another genetic cause, albeit a more complex one. The causes of breast cancer include various lifestyle factors (e.g. drinking alcohol), together with female biological sex.^14^ Neither of these ‘causes’ is necessary or sufficient (men who do not drink alcohol can get breast cancer, although it is rare, and women who do drink alcohol usually do not get the disease). However, these two causes can individually, or in combination, increase the risk of breast cancer. In Hume’s second definition, if either or both of these risk factors were absent, then some of the cases of breast cancer would not occur.

Example 3 provides a more complex (and possibly more controversial) example. In most jobs, women receive lower salaries than men. The risk of having a low salary relative to the mean for a given job is strongly affected by two factors: female sex, and sexism (institutional and/or individual). The absence of either of these factors would dramatically reduce the risk of having a low salary. Thus, both having female sex and living in a sexist society (or working for a sexist organization) are causes of lower-than-men salaries. Of course, although female sex is technically a cause in this formulation, there is a natural reluctance to describe it as such, since this may be regarded as somehow implying that it is the ‘fault’ of the group discriminated against. Thus, most would regard sexism as the fundamental cause of low salaries here (Krieger and Davey Smith give a similar argument as to why racism is the main cause of ethnic inequalities in health^32^^,^^41^). Certainly, this is the factor on which an intervention would be based (just as we intervene on diet, not PKU genes, to prevent PKU-related mental illness). Thus, the ‘causal effects’ of female sex are due to societal problems, not genetics.

Example 4 involves ‘lifestyle’ factors and obesity as causes of diabetes. Once again, both of these factors are causes of diabetes, and the risk is greatly reduced if one or both of these factors are not present.

In these pairwise lists, one would usually regard only Factor B and not Factor A as the targets for intervention (diet but not genetics, sexism but not sex etc.). Nevertheless, the states identified as Factor A are causes in the counterfactual sense. We can represent each of these states under Factor A with a variable, and if that variable is assigned a different value from its actual value, then the variable representing the outcome will also have a different value. In this sense we can say that, had Factor A been different, then the outcome would also have been different. These states may result in particular events which are themselves causes of the outcome. Thus, being female results in the proliferation of breast tissue during puberty, high adiposity causes repeated tissue inflammation, insulin resistance etc. These events are also part of the constellation of causes satisfying the condition that, had they been different, the outcome would have been different; but their inclusion in the constellation does not exclude the state which results in these events. Thus, this state is also included in the constellation of things satisfying the condition that, had they been different, the outcome would have been different. This means that states have counterfactuals (‘to be or not to be’), much as actions have counterfactuals (‘to exercise or not to exercise’).

## Distinction 2: modifiable and non-modifiable states

In the examples in Box 1, we can also distinguish between those states that we can imagine at least in principle being modifiable (dynamic states), and those that appear in principle non-modifiable (fixed states) (Table 1). For example, genetics and sex are fixed. On the other hand, obesity can be modified by exercise, diet, liposuction and other means. The distinction is not completely clear-cut, since the non-modifiability of many fixed states may be disputed (e.g. in the future, genetic modification will become increasingly possible). Thus, this distinction is not important for our general argument, rather it just affects our assessment as to whether a particular factor can currently be regarded as a fixed state or a dynamic state in a given context and time. For the purposes of the current discussion we will regard genes as fixed states, and will thus regard genetic sex as fixed, in contrast to gender identification which may change over time and can therefore be regarded as a dynamic state. Similarly, genetic ancestry (we use this term since race is an artificial construct^32^^,^^41^^,^^42^) is relatively fixed whereas ethnic identity can change over time.^42^

**Table 1.** Characteristics of different types of causes

	‘Fixed’ states	Dynamic states	Events
Examples	Sex	Gender	Smoking a pack a day
	‘Ancestry’	Ethnicity	Racism^a^
	Genetics	Racism^a^	Gene therapy
		DNA methylation	Exercise
		Obesity	Diet
		High cholesterol	Antihypertensives
		High blood pressure	
Can we explore the mechanisms?	Yes (e.g. hormonal influences on breast cancer risk)	Yes (e.g. obesity causes chronic inflammation which increases CVD risk)	Yes (e.g. effects of exercise on development of collateral vasculature and hence on CVD)
Can we make a counterfactual contrast?	Yes (e.g. genetic comparisons)	Yes (e.g. BMI = 35 vs BMI = 25)	Yes (e.g. high exercise vs low exercise)
Can we randomize?	No (e.g. sex cannot be randomized^b^)	No (e.g. obesity cannot be randomized)^c^	Yes (e.g. exercise can be randomized)
Can we intervene?	No (although we can intervene on possible mediators or take actions on intermediate states)^d^	Yes (we can carry out interventions which reduce or increase obesity)	Yes (e.g. interventions to encourage exercise)

CVD, cardiovascular disease; BMI, body mass index.

a Racism can be regarded both as a series of individual events and as a dynamic state (e.g. institutional racism).

b Sex cannot be randomized by experimenters, although in practice one can regard it ‘as good as randomized’ at conception, which is also the case of point mutations.

c Instrumental variable analysis (by Mendelian randomization) is a useful approach, e.g. by using ‘obesity genes’ to study the causal effects of obesity, but these genes are themselves causes of obesity, rather than surrogates for obesity itself.

d If sex ‘causes’ lower salaries or lower chance of tenure, one might carry out interventions on the committees that are responsible [see Hernán and VanderWeele^33^]. If BrCa1 ‘causes’ breast cancer, one might intervene by prophylactic mastectomy, hormone use, regular screening etc.

Despite these differences, for all three types of causes it is possible to specify an appropriate counterfactual contrast, and we can explore the biological mechanisms involved. Thus for all of these types of causes we can investigate causality, both in epidemiological studies and through other types of evidence. In each case, the problems of causal estimation are similar (specifying the counterfactual contrast clearly, avoiding or minimizing selection bias, information bias, confounding—in general: ruling out alternative explanations), although the relative importance of the various types of bias, and the relative importance of multiple types of evidence, may vary.

For dynamic states (e.g. obesity) events to modify them (e.g. exercise) can be randomized. Thus, for dynamic states (and for events) it is then possible to hypothesize an intervention which may or may not be fully or partially effective, whereas for fixed states intervention is not possible, even theoretically. As mentioned above, different interventions will often produce different effects: interventions to reduce blood pressure, cholesterol or obesity may have similar (but not identical) effects, and add to the evidence that high blood pressure, high cholesterol and obesity are causes of mortality. Furthermore, as Pearl^36^ argues, such interventions may have side effects which are different from the effects of the main cause under study (e.g. an intervention to reduce obesity by cycling may result in traffic accidents, which are not a result of obesity). As mentioned, the different real-life measures that are needed to stop or start ‘events’ (i.e. actions that people take) might also have different health consequences. Thus, Greenland has emphasized that the potential benefits of interventions on causes such as smoking will usually be less than might be expected if we estimate the population attributable risks of these causes.^43^

## Studying states and events as causes

After having made the above distinctions, it is useful to come back to the debate about the distinction between states and events as potential causes. It has been argued that one may not be able to draw causal inferences, or make causal estimates for states, whereas one can do so for events. However, in practice, there is no difference between these two types of causes in terms of how a study needs to be conducted and analysed. For example, consider a study which addresses two causes of incident coronary heart disease—obesity and (lack of) exercise. Suppose we were to design a cohort study following people from age 20 years and assessing their exercise rates at baseline and changes in exercise rates over time; one might compare those with (well-defined) high versus low levels of exercise, and we would collect information on all possible confounders and adjust for these. Suppose that in the same cohort study following people from age 20 years, we also assessed their BMI at baseline and changes in BMI over time; one might compare those with (well-defined) high versus low levels of BMI, and we would collect information on all possible confounders and adjust for these. Would these studies look any different, or would we analyse them in any different way? In fact, the relevant DAGs and the appropriate data analysis would be very similar (there would be some differences because the mediators might be different), and there would be the usual problems of minimizing selection bias and information bias and appropriately controlling for confounding. The fact that the former study can be conceptualized as being like a randomized trial (because exercise could be randomized), whereas the latter cannot (because obesity cannot be randomized), makes no difference in practice—and we would argue that it also makes no difference in theory.

In the causal inference literature, the discussion of whether states can be causes has often focused on the issues of consistency, when there are different possible ‘versions of treatment’. The consistency assumption requires that the exposure be defined unambiguously: using interventionist terminology, ‘one needs to be able to explain how a certain level of exposure could be hypothetically assigned to a person exposed to a different level’.^44^ In other words, is the action (or intervention) consistent across populations (or trials)? A major problem is that specifying the action (or intervention) too precisely (e.g. lifting weights at a specified weight and timing for 1 h before breakfast every morning) may make the study findings of limited use (what about other types of physical activity?). It may also make it impossible to compare across populations (or to reproduce across trials). In contrast, it has been argued^34^ that states such as obesity are not ‘well-defined’ (and therefore lack consistency). We have argued elsewhere that this criticism is largely tautological^45^—if the definition of ‘well-defined’ involves specifying a well-defined ‘intervention’, then by definition, interventions (‘actions’, ‘events’) can be well-defined whereas states cannot be. In fact, causes such as adiposity are straightforward to define and measure—we can consistently and validly measure a person’s BMI, and we can very precisely define the difference between a BMI of 35 and a BMI of 25.

VanderWeele argued in an earlier paper^46^ that states can be regarded as causes, but he added the caveat that valid causal estimation for states may be difficult, if not impossible. He argues within an interventionist framework that the quantification of the causal effect of a state remains uncertain because different interventions on a state might have different effects, i.e. there are multiple possible interventions, and the causal effect of the state (a ‘composite cause’^46^) is estimated by some function of the individual causal effects of these multiple interventions. However, as we have argued above, and elsewhere,^30^ obesity is not a ‘composite exposure’. Rather it is a single exposure which causes mortality; it can be caused by multiple factors, and can be reduced by multiple interventions. Of course, BMI can be changed in ways (e.g. by cutting someone’s arms off) that do not directly affect obesity-related diseases. However, this just means that BMI is a poor measure of adiposity, which is the underlying state that causes obesity-related diseases.^47^ More generally, it could be argued that different types of obesity carry different mortality risks, and that we should study more specific subtypes of obesity; but this applies to most exposures. We are always estimating average population effects, for example the average effect on mortality of a BMI of 35 versus 25, or the average effect on mortality of smoking 10 pack-years versus 0 pack-years. This does not depend on specifying interventions.

One final distinction should be mentioned. Some states have considerable advantages when we are doing causal inference. Some fixed states (e.g. being of male or female sex, having a particular genetic mutation) have few, or even no, potential confounders (for example, we can easily study the different life courses of boys and girls born into the same family, or of siblings with different genetic profiles). Thus, we can often estimate the causal effects of these states with more confidence and validity than is often the case when studying events as causes. Rather than states being impossible to study, in terms of causal estimation, in some instances, they are much easier to study.

There are several ways in which an estimate contrasting people with different levels of a state is useful. It can be seen as a form of aetiological causality. For example, contrasting the mortality between levels of serum cholesterol or blood pressure estimates the amount of disease that can be ascribed to these differences. Also, in contrasting the incidence of breast cancer between BrCa1 carriers and non-carriers, the amount of disease due to the gene is estimated.

Of course, as noted above, although it is possible to estimate an overall effect of a state on disease occurrence, different actions on a state will have different effects; different ways of treating hypertension or hypercholesterolaemia (e.g. lifestyle, different drugs etc.), will have different effects—but that does not detract from the causality of hypertension or hypercholesterolaemia. Likewise, different actions on persons who are carriers of BrCa1 (frequent screening, prophylactic mastectomy, oophorectomy) will have different actual effects, and will also yield estimates that are different when contrasting carriers and non-carriers for the occurrence of breast cancer. This is not different from what we mentioned above, i.e. that different measures to stop people smoking may have different effects.

## Why does this matter?

In our view, considering states as causes, on equal footing with events, is important because it enables investigators to identify important scientific and public health problems. We are only involved in research into the prevention and reduction of hypertension, obesity etc. because epidemiological studies have established these conditions as causes of disease, and there is good biological knowledge about the likely mechanisms.^48^ Thus, Pearl^36^ wrote:

Why do physicians communicate with each other through these measurements, instead of through the ‘interventions’ that may change these measurements? The reasons lie, again, in the scientific meaning of these entities and their stability across domains.

States such as obesity have been considered as causes of cancer by the International Agency for Research on Cancer,^48^ and their causal effects can be studied not only in ‘standard’ observational studies, but also using techniques such as Mendelian randomization which has the added benefit of defining an ‘tendency towards obesity’ that existed at birth, i.e. before middle-aged obesity actually developed.^49^

Once it has been established that a state is (or is likely to be) a cause, this then produces a cascade of research into the mechanisms involved (aetiological causality), and this in turn leads to interventions (interventional causality) to prevent disease by modifying these states. Furthermore, in the example of obesity, epidemiologists have produced quantitative estimates of its causal effects.^48^ The quantification of the causal effect of a state serves two purposes: it shows how much of the disease is due to that state (after excluding confounding and other biases), and thus how important the state is in public health terms. As such, it shows whether the state can become a worthwhile object of action. It helps to give an explanation of how a particular disease comes about. It is included in a narrative chain about events, interacting with each other and the wider environment, from which can be gleaned which interventions at what point might be most feasible.

The fact that states can be causes is relevant to many common medical and public health questions. NIHCE guidelines for the treatment of hypertension (a state) make different recommendations depending on age and race/ethnicity (also states).^50^ Furthermore, often states become the subject of feasible interventions through further research. Hypercholesterolaemia was held to be a cause of cardiovascular diseases, long before effective treatment existed^51^; several early attempts at lowering cholesterol by drugs, exercise or diet proved not very effective, but that did not deter epidemiologists from continuing to see elevated cholesterol as a cause, which was vindicated with the advent of statins.^51^

## Discussion

The purpose of this paper has been to clarify how considering different types of causes and different types of causality is necessary in epidemiological practice. Worthwhile investigations into causality are of two types: explanatory investigations and interventionist investigations.^8^ Causes can be regarded as events or states, and the latter can be regarded as dynamic or fixed. Furthermore, causation can be considered at a number of different levels.^4^^,^^5^ All of these types of causes that exist at different levels are susceptible to causal investigation, and whatever type of cause we are studying, we need to consider the usual problems of selection bias, confounding etc. However, the nature of the evidence involved (e.g. whether or not it is possible to do a randomized controlled trial, or an observational study that closely mimics one), and the level and type of auxiliary evidence^14^ required (e.g. whether mechanistic evidence is available), may differ according to the type of cause under study and may be highly context-specific. Although the possible issues that one has to consider (such as biases) differ to some extent when studying these different types of causes, there are substantial commonalities. Every process of causal identification and explanation involves a variety of evidence, and usually no single study is definitive.

## Funding

The research leading to these results has received funding from the European Research Council under the European Union’s Seventh Framework Programme (FP7/2007–2013)/ERC grant agreement no 668954, and from the UK Medical Research Council (MR/P02386X/1).
